# Molecular dynamic simulations of oxidized skin lipid bilayer and permeability of reactive oxygen species

**DOI:** 10.1038/s41598-019-40913-y

**Published:** 2019-03-14

**Authors:** Dharmendra Kumar Yadav, Surendra Kumar, Eun-Ha Choi, Sandeep Chaudhary, Mi-Hyun Kim

**Affiliations:** 1 Gachon Institute of Pharmaceutical Science & Department of Pharmacy, College of Pharmacy, Gachon University, Incheon, 406-799 South Korea; 2 Plasma Bioscience Research Center/PDP Research Center, Kwangwoon University, Nowon-Gu, Seoul, 139-791 Korea; 3 Laboratory of Organic and Medicinal Chemistry, Department of Chemistry, Malaviya National Institute of Technology, Jaipur, 302017 India

## Abstract

Lipid peroxidation by reactive oxygen species (ROS) during oxidative stress is non-enzymatic damage that affects the integrity of biological membrane, and alters the fluidity and permeability. We conducted molecular dynamic simulation studies to evaluate the structural properties of the bilayer after lipid peroxidation and to measure the permeability of distinct ROS. The oxidized membrane contains free fatty acid, ceramide, cholesterol, and 5α-hydroperoxycholesterol (5α-CH). The result of unconstrained molecular dynamic simulations revealed that lipid peroxidation causes area-per-lipid of the bilayer to increase and bilayer thickness to decrease. The simulations also revealed that the oxidized group of 5α-CH (-OOH) moves towards the aqueous layer and its backbone tilts causing lateral expansion of the bilayer membrane. These changes are detrimental to structural and functional properties of the membrane. The measured free energy profile for different ROS (H_2_O_2_, HO_2_, HO, and O_2_) across the peroxidized lipid bilayer showed that the increase in lipid peroxidation resulted in breaching barrier decrease for all species, allowing easy traversal of the membrane. Thus, lipid peroxidation perturbs the membrane barrier and imposes oxidative stress resulting into apoptosis. The collective insights increase the understanding of oxidation stress at the atomic level.

## Introduction

The largest organ of the human body i.e. skin which acts as an environmental interface and are continuously subject to exposure to chemical mutagens and carcinogens, either accidently or occupationally^1^. Skin cancer is a major and growing public health problem, that accounts for approximately 40% of all new cancer diagnoses^2^. The majority (approximately 80%) of skin cancers are basal cell carcinomas (BCC), with squamous cell carcinoma (SCC) and melanoma accounting for approximately 16% and 4%, respectively^3^. BCC and SCC are non-melanomas that originate from epidermal keratinocytes. They are associated with chronic sun exposure, whereas melanoma originates from melanocytes and is associated with intermittent sun exposure^4–6^.

The stratum corneum (SC), being the outermost layer of the skin is remarked to impart the barrier capacity to the skin. The SC is a structured molecular organization of corneocytes (brick) embedded in a lipid matrix (mortar) which possess the potential for non-invasive permeability^7,8^ and that imparts the barrier functions. The lipid matrix of SC is composed of mixture of long-chain ceramide (CER-NS), cholesterol (CHO), and free fatty acids (FFAs) in almost equal molar ratio (1:1:1)^9–11^. Skin exposure to ionizing or ultraviolet (UV) radiation, metal-catalyzed reactions, inflammation-mediated damage to neutrophils and macrophages, and mitochondria-catalyzed electron transport reactions produces the free radicals generally referred to as reactive oxygen species (ROS) and reactive nitrogen species (RNS)^12–15^. ROS, which include superoxide anion (O_2_^−^), hydrogen peroxide (H_2_O_2_), singlet oxygen (^1^O_2_), and hydroxyl radicals (HO•), are continuously generated at low levels during the course of normal aerobic metabolism^16,17^. Membranes of skin cells contain antioxidant defenses to nullify excessive ROS produced during exposure. However, chronic exposure to ROS can over whelm the antioxidants and other oxidant-degrading pathways^18^. The skin lipid membranes oxidized by a different mechanism and among them, the non-enzymatic process of photo-oxidation is prevalent^19^. Photo-oxidation that leads to lipid peroxidation has significant effects on the structure and dynamics of lipid membranes, which include increased water permeability, decreased lipid bilayer thickness, or alterations in the lipid membrane order and fluidity. These alterations produce peroxidative stress in the membranes^20–22^. Under oxidative stress, relatively stable and prominent lipid hydroperoxide (LOOH) intermediates form and accumulate within the skin lipid bilayer membranes that results in altered structural organization and packing of bilayer lipid components^23–25^. The peroxidation process targets the polyunsaturated components of lipid bilayer of skin and cholesterol as one of the prime targets, rapidly undergoes peroxidation to give rise of hydroperoxycholesterol (ChOOH)^24–26^. Hydroperoxycholesterol consists of 5α-ChOOH, 6α-ChOOH, and 6β-ChOOH^23,26^. CHO is abundant in mammalian cells and tissues and well known for maintaining the integrity, permeability, fluidity, and phase behavior of membrane bilayer. CHO is also believed to disseminate the stress due to lipid peroxidation. Among all the hydroperoxycholesterol products, 5α-ChOOH (5α-CH) is considered to alter membrane structure which follows perturbed barrier function causing inflammatory responses and carcinogenesis^27,28^.

Herein, we examined as to how degree of lipid peroxidation influence the biomembrane properties and permeability of the ROS i.e. H_2_O_2_, HO_2_^•^, HO^•^, and O_2_ through the oxidized lipid bilayer of skin following an umbrella sampling (US) method by the calculation of the potential mean of force (PMF). It has been studied that the ROS interacts with those hydrophilic groups of the lipid bilayer, which are more prone to undergo oxidation and that causes peroxidation of the lipids. Such peroxidation would alter the structural and dynamic properties of the membrane^29,30^. These experimentally observed membrane properties computationally rationalized and reported in several studies. Likewise, a computational approach demonstrated influences on lipid membrane properties^31^ due to phospholipid and cholesterol peroxidation. Furthermore, a non-reactive molecular dynamic simulation (MDS) study following a single component of the phospholipid bilayer exhibited relatively thin, loosely packed and disordered membranes due to lipid peroxidation which resulted in enhanced permeability^32^. Simulations incorporating the skin-lipid bilayer comprised of CER NS (24:0), FFAs (24:0), and CHO were performed and subsequently calculated the permeability and diffusivity of water molecules^33,34^. Similarly, constrained united atom molecular dynamic simulation computed the permeability of different drugs and solutes across skin-lipid bilayer membrane^35,36^. Furthermore, our group has carried out a MDS work on native skin-lipid bilayer membrane and calculated the permeability of various ROS^37^. However, no simulation work has focused on oxidized skin-lipid bilayer and the permeability of ROS.

Since, cholesterol a major component of the skin-lipid bilayer along with other CER and FFAs confers fluidity and rigidity to membranes. Thus, lipid peroxidation may deplete cholesterol, leading to a weakened membrane that is more susceptible to oxidative stress. Studies reported that, the decrease in cholesterol content in the native skin-lipid bilayer below 50 mol%^38–41^ causes rise in oxidative stress. Previously, we have modeled two oxidized systems comprised of CER + CHO + FFA, with 12.5 and 50 mol% of 5α-CH (hydroperoxycholesterol)^42^. The present work extends these findings to computationally model two additional oxidized systems comprised of 25 and 60 mol% of 5α-CH and measure the transfer free energy of ROS, with the goal of establishing an understanding between degree of peroxidation and permeability of ROS across the skin-lipid bilayer membrane.

## Results and Discussion

### Analysis of membrane properties

**Figure 1:**
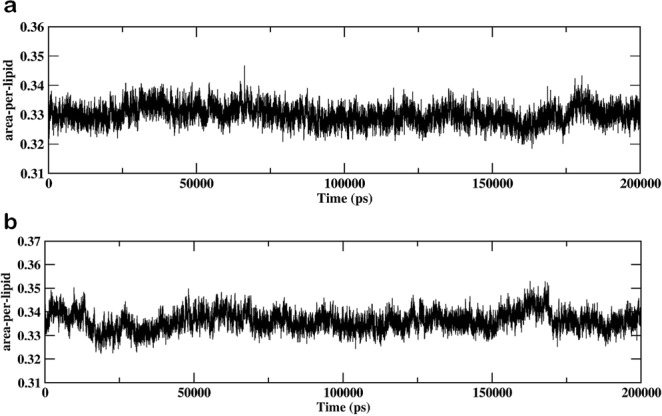
Evolution of area per lipid for the (**a**) MEMB_25 system and (**b**) MEMB_60 system.

The influence of hydroperoxycholesterol in oxidized skin-lipid bilayer membranes was investigated by calculating the various membrane properties. The APL for oxidized skin-lipid bilayer (25 mol% of 5α-ChOOH (MEMB_25) and 60 mol% of 5α-ChOOH (MEMB_60)) was calculated and compared (Fig. 1). The APL was 0.329 ± 0.003 nm for MEMB_25 and 0.338 ± 0.004 nm for MEMB_60. The observed higher value for MEMB_60 corroborates previous experimental and computational results^32,43,44^ of the increased APL of lipid bilayers upon oxidation or increased degree of oxidation. Our previous finding of APL for native (un-oxidized) skin-lipid bilayer was 0.32 ± 0.003 nm, and upon comparison with which further supports our computational finding that, peroxidation, increases APL^37^.

**Figure 2:**
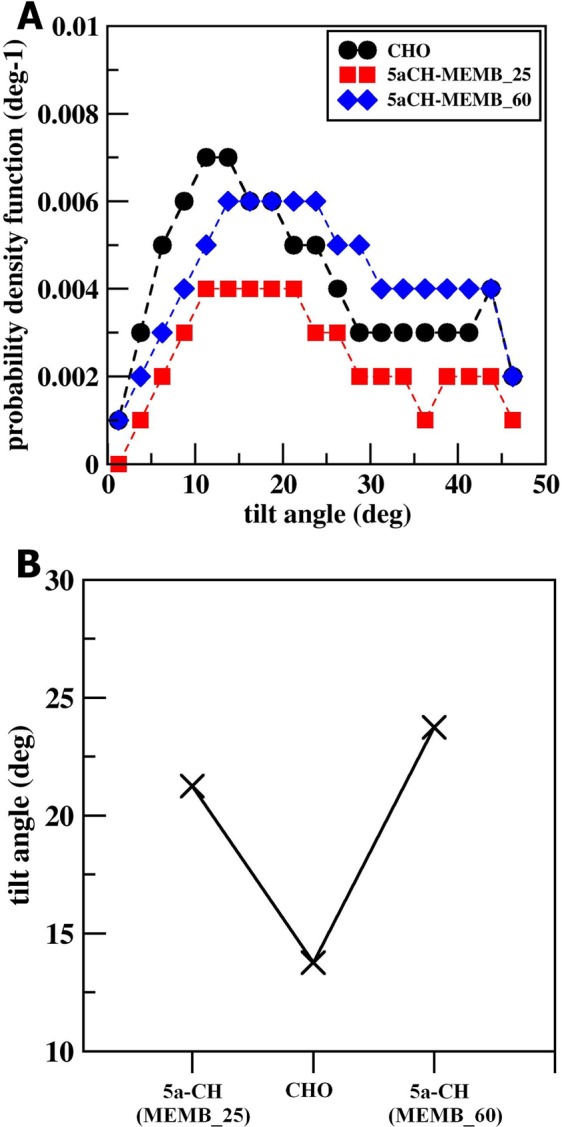
(**A**) Tilt angle distribution of the sterol backbone of CHO, 5α-CH in MEMB_25 and MEMB_60. (**B**) Tilt angles of cholesterol (CHO), 5α-CH in MEMB_25 and MEMB_60.

**Figure 3:**
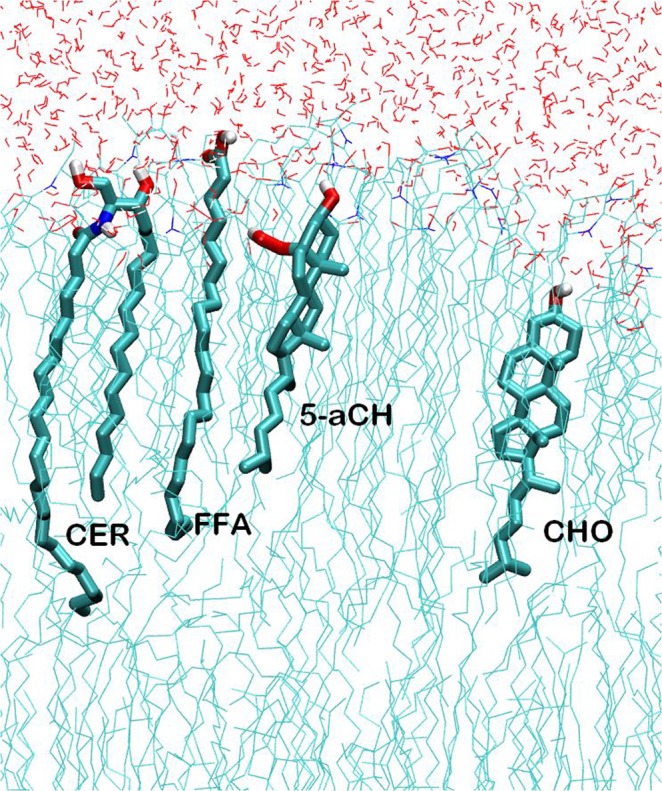
Selected lipids in “CER + CHO + FFA + 5α-CH” systems. Atoms are represented in white (H), cyan (C), blue (N), and red (O).

Likewise, it has been observed previously that peroxidation of lipid bilayers has also significant effects on bilayer thickness^43,44^. Therefore, we calculated the thickness of the bilayer during the last 50 ns simulation. The values were 4.76 nm and 4.52 nm for MEMB_25 and MEMB_60 respectively. These results suggest that an increase in oxidized components in skin-lipid bilayer membranes results in the decrease in bilayer thickness. We further calculated the tilt angle of CHO and 5α-CH in both oxidized skin-lipid bilayer membrane and found the increase in tilt angle for 5α-CH as degree of oxidation increases (Fig. 2). The increased tilt, may be considered as due to oxidation of CHO components (5α-ChOOH), the polar group (-OOH) heads move towards the aqueous layer for enhanced interaction with water molecules and tilting of backbone in 5α-ChOOH occupy the extra volume that brings about lateral expansion of membrane (Fig. 3). This bended conformation results in greater APL and decreased membrane thickness within oxidized systems. This corroborates earlier findings^31^.

**Figure 4:**
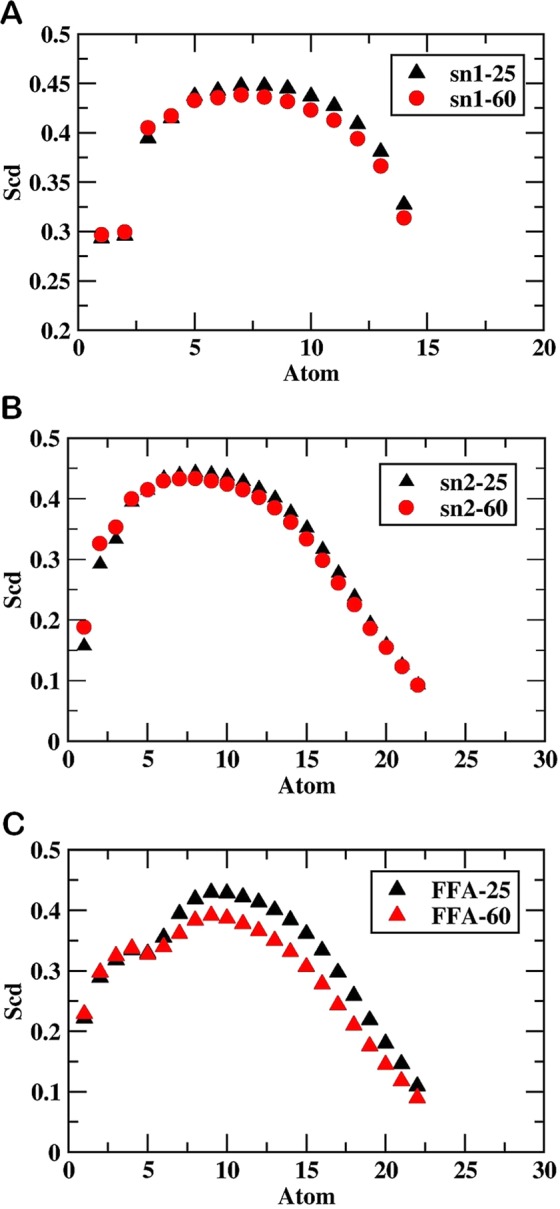
Values of order parameter S_CD_ along: (**A**) sn1 of MEMB_25 and MEMB_60, (**B**) sn2 of MEMB_25 and MEMB_60, and (**C**) lipid tail of FFAs.

The influence of oxidized components then examined over the order parameter of the lipid tail. The tail order parameters for hydrophobic chains of CER and FFA were calculated in both the oxidized systems (Fig. 4). The sn1 and sn2 lipid tail of CER showed low order parameters near C16 and C24, respectively. In addition, it increased with progression to the middle of the bilayer, and followed by a decrease towards the middle of the bilayer (Fig. 4A,B). Similarly, in the presence of oxidized components, the order parameters for FFA chain length followed a similar trend as that for the CER (Fig. 4C). The nearly identical profiles observed for both systems, however, MEMB_60 represented lower tail order parameters to that of MEMB_25. This could be interpreted as, the presence of polar (-OOH) groups in 5α-ChOOH caused distortion in the bilayer which could be distinctly marked in MEMB_60.

**Figure 5:**
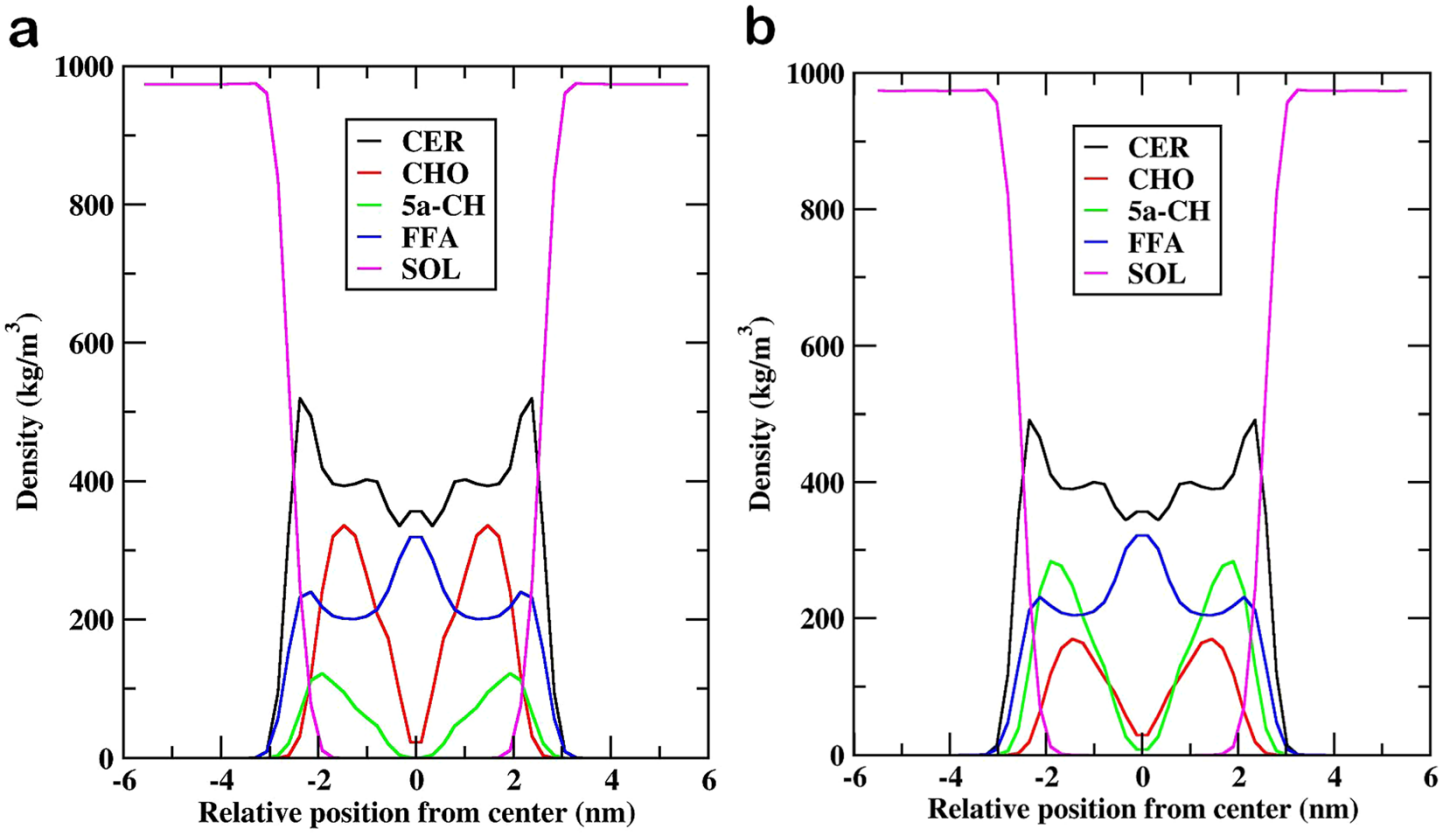
Density profile of individual lipid components (CER; CHO; FFA; 5α-CH) and water (SOL) for (**A**) MEMB_25 and (**B**) MEMB_60 along the bilayer normal (z).

Similarly, for both the systems, the densities of individual lipid components have been plotted across the bilayer normal (z-axis) (Fig. 5). In both oxidized systems, a constant density was noticed from z ~ −6 nm to z ~ −3nm that corresponds to bulk water. There was a decrease in the density of water as moved close to interface, and then density increased for headgroups of all the lipids (z ~ −3.0 nm to z ~ −1.5 nm). The skin lipid barrier properties could be affirmed due to the observed density for the tightly packed lipid tails in the region from z ~ −1.5 nm to z ~ −0.4 nm. The disordered packing of lipid tails was comprehended as the density was minimal at z ~ 0. The CER density manifested as a sharp peak near the lipid-water interface below which settled all individual lipid headgroups. The CHO and 5α-CH settled in the high-density region of the bilayer owing to its small size and low partial charge of their head groups as observed from density profile. Furthermore, the ordered interdigitation of lipid tails manifested as a small peak at the bilayer center in the CER and FFA density profile, which was not observed for CHO and 5α-CH being small size and shorter chain length.

### Effect of ROS on membrane permeability

**Figure 6:**
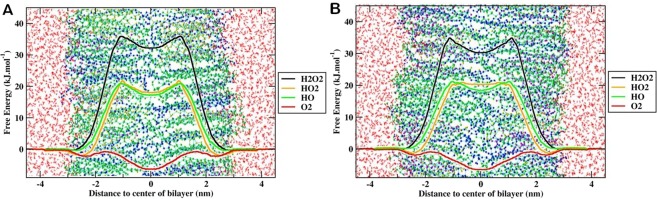
Free energy profile of different reactive oxygen species (ROS) across oxidized skin-lipid bilayer (CER-CHO-FFA-5α-CH) membrane (**A**) MEMB_25, and (**B**) MEMB_60.

We further measured the transfer FEP for ROS through the membrane of MEMB_25 and MEMB_60 by averaging the PMFs for all ROS and presented in Fig. 6 and Table 1. The results described in detail in the following subsections.

**Table 1:** Transfer free energies (ΔG) of all investigated ROS in the oxidized skin-bilayer lipid (CER-CHO-FFA-5α-CH) membrane.

Oxidized Skin-lipid Bilayer System	ROS	ΔG(kJ/mol)
MEMB_25	H_2_O_2_	36.01 ± 1.33
	HO_2_	23.69 ± 1.87
	HO	25.01 ± 1.19
	O2	−5.91 ± 0.66
MEMB_60	H2O2	35.25 ± 1.37
	HO_2_	21.65 ± 1.76
	HO	21.51 ± 1.17
	O2	−5.56 ± 0.87

The standard errors are derived from the transfer free energies of all 33 independent profiles.

**Table 2:** Composition of the simulated skin-lipid bilayer membrane systems.

Oxidized Skin-lipid Bilayer System	Composition			
	CER	CHO	FFA	5α-CH
MEMB_25 (25 mol% of 5α-ChOOH)	52	38	52	12
MEMB_60 (60 mol% of 5α-ChOOH)	52	20	52	30

The total number of lipid molecules was 154 in each bilayer system with 77 in one of the leaflet and 77 in the other.

### Transfer free energy profile of ROS in the MEMB_25 system

The transfer free energy in terms of the PMF for all ROS across the oxidized skin-lipid bilayers is shown in Fig. 6(A). In the aqueous phase, the free energy barrier for H_2_O_2_, HO_2_^•^, HO^•^, and O_2_ were low or nearly negligible. However, as the ROS reached the vicinity of the headgroups of the lipid bilayer (z ~ 3 nm), a significant decrease in the free energy for H_2_O_2_, HO_2_^•^, and HO^•^ species was observed which could be attributed to the interaction of partially charged and hydrophilic headgroups. For O_2_, we observed a slightly increase (~0.23 kJ/mol) in free energy. These observations are in accordance with the transfer free energy of hydrophilic and hydrophobic species, where the free energy for hydrophilic species is slightly decreased and is slightly increased for hydrophobic species^36^. Furthermore, the comparison of the transfer free energy of hydroperoxyl (HO_2_^•^) and hydroxyl species (HO^•^), HO_2_^•^ displayed greater interaction in the headgroups region that may be due to the additional oxygen atom, and greater van der Waals interactions with headgroups of the lipid bilayer. Furthermore, HO_2_^•^ species are better proton donors and weaker acceptors. On the other hand, HO^•^ species remains good H-bond donor/acceptors than water, which help them interact suitably with lipid headgroups^42,45^. The experimental study is in accord with current observations relating to the permeability of various ROS^46^. There was an enhancement in the transfer free energy after head group region while being maximum in the hydrophobic core for H_2_O_2_ (~36.01 kJ/mol), HO_2_^•^ (~23.69 kJ/mol), and HO^•^ (~25.01 kJ/mol) which then decreased to respective values of (~32.26 kJ/mol), (~19.46 kJ/mol), and (~25.01 kJ/mol) at the center of the bilayer. However, due to hydrophobic nature of the O_2_ species, its PMF profile was distinct from those of other types of ROS. For O_2_ species, the transfer free energy beyond the headgroups region of the lipid bilayer decreased to ~−1.76 kJ/mol and then increased to ~0.11 kJ/mol. The observed new energy barrier for O_2_ species (at ~1.5 nm) corresponds to the ring structure of CHO and 5α-CH in the oxidized skin-lipid bilayer membrane. Moving further in membrane, the O_2_ species has displayed energy minima (~−6.01 kJ/mol) at the bilayer center corresponds to the absence of lipids in the center. The average transfer free energy in 33 independent simulation result is shown in Table 1. The comparison of transfer free energy barriers for all ROS revealed that the permeation of H_2_O_2_ was most hindered with an estimated free energy of 36.01 ± 1.33 kJ/mol, while O_2_ was least hindered with an estimated free energy of-5.91 ± 0.66 kJ/mol.

### Transfer free energy profile of ROS in the MEMB_60 system

Figure 6(B) shows the transfer free energy of ROS across the oxidized skin-lipid bilayer. Its PMF profile was equivalent to the PMF profile of MEMB_25. Likewise, a decrease in transfer free energy observed as ROS reached the lipid bilayer headgroups (z ~3 nm). The transfer free energy increased upon the entry of ROS in the hydrophobic region and reached maximum in the high-density region of the oxidized skin-lipid bilayers. As the ROS moved further, the transfer free energy decreased to a minimum around the center of the bilayer. The approximate maximal transfer free energies for H_2_O_2_, HO_2_^•^_,_ HO^•^, and O_2_ were 35.25, 21.65, 21.51, and 1.33 kJ/mol, respectively. The approximate minimal values in the same respective order were 31.01, 20.22, 18.46, and −6.90 kJ/mol. The calculated average transfer free energy in the 33 independent simulation result is shown in Table 1. Similarly, upon comparison the transfer free energy barriers for ROS in the MEMB_60 system revealed that H_2_O_2_ was least permeant with a free energy of 35.25 ± 1.37 kJ/mol, while O_2_ was more permeant with a free energy of −5.45 ± 0.85 kJ/mol.

The present work, computationally modeled the MEMB_25 and MEMB_60 oxidized skin-lipid bilayer systems differs in the number of oxidized components (5α-ChOOH). The studied membrane properties from MDS revealed that as the degree of peroxidation of skin-lipid bilayer increased, the APL also increased and the bilayer thickness of the membrane decreased, which led to lateral expansion of the membrane. This lateral expansion represent the adjustment of polar groups (-OOH) of 5α-CH in the headgroups region of the lipid bilayer. Thus, these structural alterations resulted in a relatively thin, loosely packed and disordered system that is vulnerable to damages by ROS.

Furthermore, upon comparison of the calculated transfer free energy values for all ROS in MEMB_25 and MEMB_60 membrane systems demonstrated that increased oxidized components (5α-CH) in the skin-lipid bilayer diminished the resistant properties of the membranes and decreased the transfer free energy value. Thus, ROS would more easily be able to breach the transfer free energy barriers and permeate across the membrane, causing oxidative stress, which may result in apoptosis.

## Conclusion

Non-enzymatic lipid peroxidation occurs in skin-lipid bilayer membranes due to photo-oxidation. It hampers the structural organization of the membrane by changing lipid packing and the thermodynamic and phase parameters. These structural changes are responsible for perturbations in skin-lipid barrier, inflammatory responses, and carcinogenesis. The present study related the degree of peroxidation product (hydroperoxycholesterol) with membrane properties and calculated the transfer free energy of ROS across two different oxidized skin-lipid bilayers using united-atom MDSs. The computationally model MEMB_25 and MEMB_60 membrane systems were constituted of CER, CHO, FFAs, and 5α-CH. The studied membrane properties included APL, bilayer thickness, tail order, and density profile of the individual lipid components. The results revealed that the degree of peroxidation significantly affects the membrane properties, which include increased APL, decreased bilayer membrane thickness, and the tail order parameter. It was observed that the polar groups (-OOH) of oxidized components (5α-CH) undergo tilt in skin-lipid bilayer membrane during simulation and orient toward the aqueous phase along the bilayer normal, which causes the bilayer membrane to expand laterally and further disorder the integral structure of the membrane. We further calculated the transfer free energy of ROS through the oxidized skin-lipid bilayer membranes. The transfer free energy profile for the MEMB_25 and MEMB_60 systems indicated that free energy barrier for all ROS decreases as the degree of peroxidation increases. Similarly, we observed lower transfer free energy for MEMB_60 as compared to MEMB_25, which indicates that peroxidation of lipid components in the skin-lipid bilayer, has significant effects on lipid barrier properties. Thus, lipid peroxidation causes perturbational changes in skin-lipid bilayer membrane that facilitates the easy transport of all ROS along the lipid bilayer. The breaching of the free energy barrier at low transfer energy would make ROS to permeate the membrane, whereby the consequent oxidative stress may cause apoptosis.

## Simulation Methods

### Composition and description of the model systems

**Figure 7:**
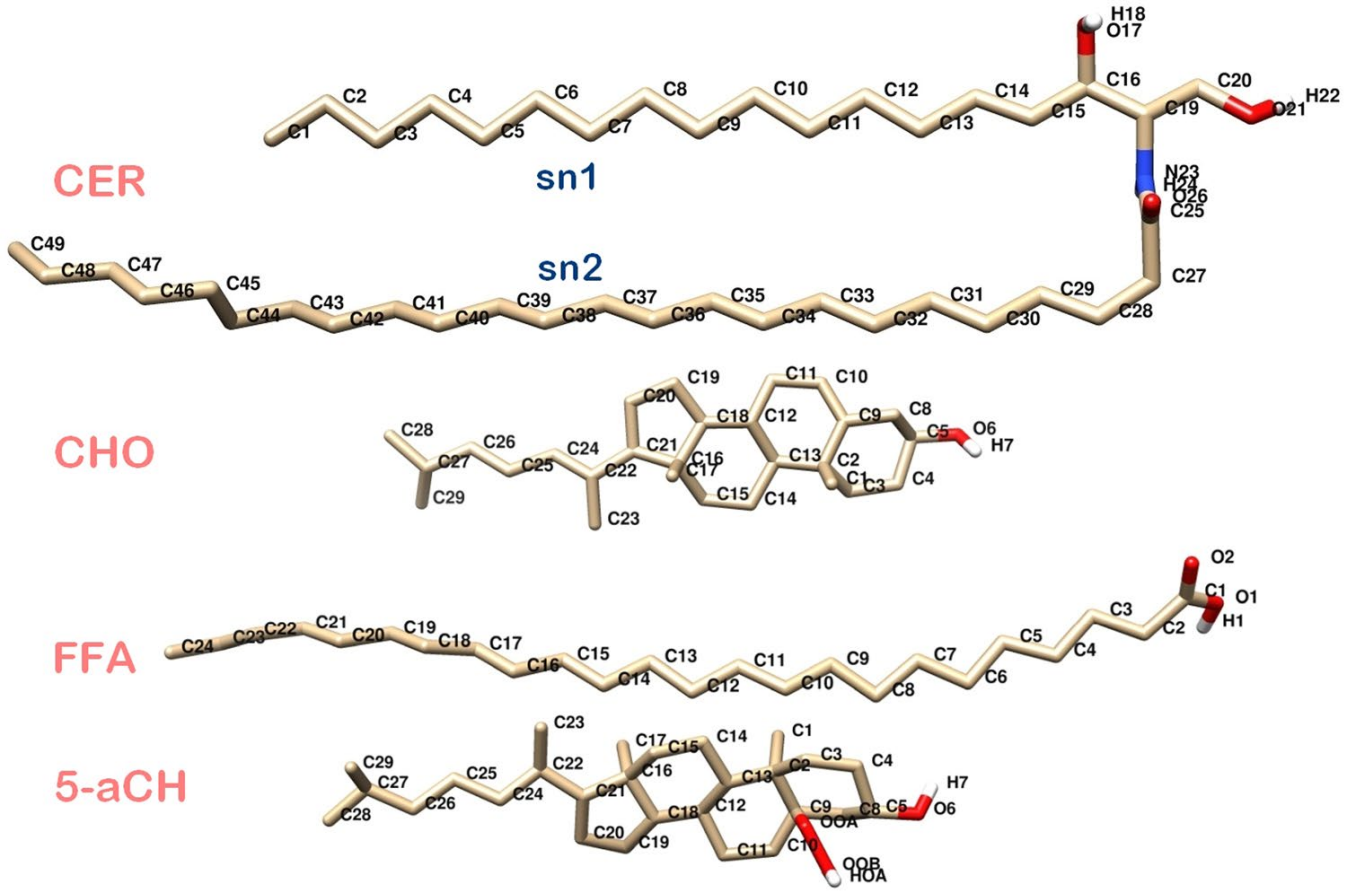
Molecular structure of individual lipids, i.e. Ceramide NS (CER), Cholesterol (CHO), Free fatty acid (FFA), and 5α-ChOOH (5α-CH) used in simulations. Oxygen, hydrogen, carbon, and nitrogen atoms are shown in red, white, tan, and blue, respectively.

The oxidized skin-lipid bilayer membrane constructed in this work contains CER-NS (C24:1), FFA (C24:0), and CHO along with hydroperoxycholesterol (the oxidized form of CHO). Hydroperoxycholesterol in each system constitutes 25 and 60 mol % of CHO. Thus, each oxidized system is a heterogeneous mixture of 154 individual components (Fig. 7 and Table 2).

We have opted the same methodology as reported by our group to construct the oxidized skin-lipid bilayer membrane system^47^. The force field parameters for CER, CHOL,and FFAs were based on prior studies^48,49^. For 5α-CH, the parameters were taken from reported work of Neto, *et al*.^31^. The simple point charge (SPC) model was used for the water molecules^37^. The parameters of the ROS have been previously described^50^.

All the simulations performed under NPT ensemble using the GROMACS 5.1.4 MD package^51–54^. The simulation conditions were taken from the previously reported work^35,37^, however in brief the temperature was controlled at 310.15 K by a Nose-Hoover thermostat with a time constant of 0.5 ps and coupled separately to lipid and water molecules. The pressure was applied at 1 bar using a Parrinello-Rahman barostat with a time constant of 5 ps and compressibility of 4.5 × 10^−5^ bar with semi-isotropic coupling. A time steps of 2 fs used for all simulations. The cut-off distance for columbic interactions and van der Waals interactions were both set at 1.2 nm. The periodic boundary condition was applied in all three directions.

The build oxidized skin-lipid bilayer was first energy minimized using the steepest descent algorithm followed by NVT equilibration for 2 ns under restrained conditions. Further, the equilibrated bilayer simulated for 10 ns in the NPT ensemble prior to simulated annealing, where the system heated to 360 K and then cooled to 310.15 K in a systematic manner to obtain well-hydrated lipid bilayer heads. The system further equilibrated for 50 ns, followed by 200 ns production simulation under the NPT ensemble conditions. Furthermore, the structure obtained (ESI, Fig. S1) used to calculate the various membrane properties, that includes area per lipid (APL), bilayer thickness, density distribution of individual components, tail order parameters and tilt angle of backbone of cholesterol and 5α-CH with respect to membrane normal (z-axis). Moreover, we have further estimated the permeability of the various ROS in the two different oxidized skin-lipid bilayer systems.

### Analysis of membrane properties

In order to understand the effect of degree of lipid peroxidation product on the membrane properties of bilayers, the aforementioned parameters were calculated. Area per lipid (APL) describes the packing of a lipid bilayer and in an oxidized skin-lipid bilayer system, the addition of the hydroperoxycholesterol lipid component might have an overall impact on the APL. Several methods described the calculation of APL^55–57^. However, in the present analysis, we have used the previously described method for its calculation during the final 50 ns of the simulation^37^. Similarly, the membrane thickness an important parameter that influence the permeability of charged or neutral molecules, and plays a role in unfolding the properties of different oxidized system was measured using APLVORO software^58^ on the last 50-ns simulation trajectory. During the calculation, the key atoms (O21 for CER, O6 for CHO and5α-CH, and O2 for FFA) from headgroups of each lipid component were defined and average position between each headgroups of both leaflets were used to measure the membrane thickness. Furthermore, the density distribution of each lipid components in oxidized skin-lipid bilayer was computed to investigate its arrangement along the z-direction^37^. Finally, the orientations of lipid hydrocarbon in the bilayer were measured by calculating the tail order parameters^37,42^. It represent the average value of the deuterium order parameter (S_CD_)^59^, and is calculated as follows:

$${S}_{CD}=\frac{1}{2}\langle 3{\cos }^{2}({\theta }_{j})-1\rangle $$

Where, θj is the angle between a C-H bond of the ith carbon and the bilayer normal (z axis). The angular brackets show an ensemble average. If the value of S_CD_ equals 1, the lipid tails are perfectly oriented along the z-axis, while a value of −0.5 indicates an orientation perpendicular to the bilayer normal^37,42^.

### Transfer free energy profiles

The US methodology adopted to calculate the transfer free energy profiles (FEPs) of each ROS molecules across the oxidized skin-lipid bilayer membrane in order to understand the hydroperoxycholesterol concentration-dependent transfer FEP changes. We have followed the same methodology from our previous work to perform the US^37,42^. The equilibrated structure as shown in (ESI Fig. 2) taken as input and for each ROS, a total of 33 systems were created. Each system (Membrane + ROS) was energy minimized and then equilibrated, while keeping the ROS molecules fixed at their current position under NPT ensemble. Each US simulation lasted 20 ns with the final 10 ns used for analysis, i.e., to acquire the US histograms and calculate the FEPs. The weighted histogram analysis method (WHAM) as implemented in GROMACS was used to construct the FEPs^60^. In order to improve the statistical accuracy of sampling, FEPs obtained by averaging six FEPs for each ROS.

## Supplementary information

Supplementary Dataset
