# Supplementary material for: Molecular dynamic simulations of oxidized skin lipid bilayer and permeability of reactive oxygen species
Source: Sci Rep. 2019 Mar 14;9:4496. doi: 10.1038/s41598-019-40913-y (PMC6418262; doi:10.1038/s41598-019-40913-y)

**SUPPLEMENTARY DATA**

**Molecular​ ​dynamic simulations of​ ​oxidized skin lipid bilayer and permeability of reactive oxygen species**

Dharmendra Kumar Yadav^a,$*^, Surendra Kumar^a,$^, Eun-Ha Choi^b^, Sandeep Chaudhary^c^, Mi-Hyun Kim^a*^

^a^Gachon Institute of Pharmaceutical Science & Department of Pharmacy, College of Pharmacy, Gachon University, Incheon, 406-799, South Korea.

^b^Plasma Bioscience Research Center/PDP Research Center, Kwangwoon University, Nowon-Gu, Seoul

139-791, Korea

^c^Laboratory of Organic and Medicinal Chemistry, Department of Chemistry, Malaviya National Institute of Technology, Jaipur, 302017 India.

*Email:* [*dharmendra30oct@gmail.com*](mailto:dharmendra30oct@gmail.com)*, k*[*mh0515@gachon.ac.kr*](mailto:mh0515@gachon.ac.kr)

*corresponding author

$contributed equally

**Dr.Dharmendra Kumar Yadav, Ph.D**

Research Professor

Office: +82-32-820-4947

Email: [dharmendra30oct@gmail.com](mailto:dharmendra30oct@gmail.com)

**Figure S1.** Oxidized skin-lipid bilayer **structure containing (A) 52 CER (Green), 38 CHO (Yellow), 52 FFA (blue), 12 5α-CH (Magenta) molecules and 5210 water molecules; (B) 52 CER (Green), 20 CHO (Yellow), 52 FFA (blue), 30 5α-CH (Magenta) molecules and 5210 water molecules. Headgroups are shown in pink sphere (VDW).**


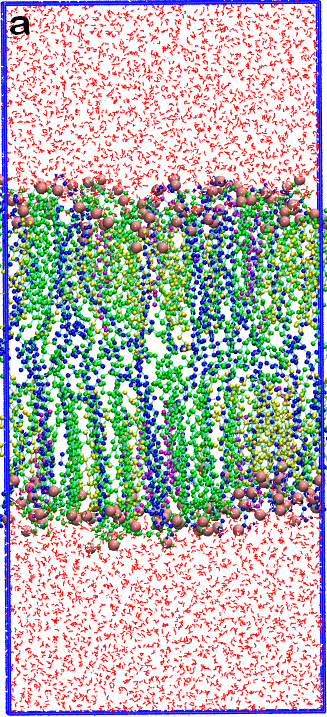

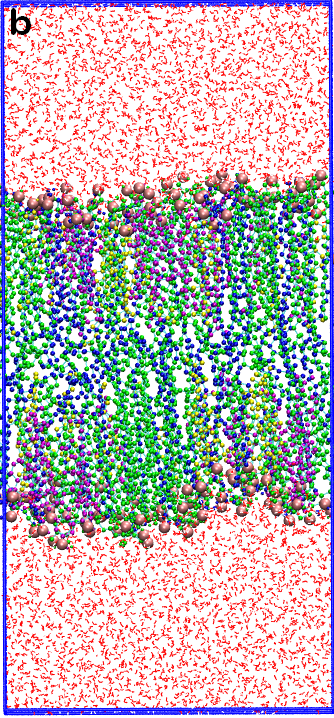


**Figure S2.** Illustration of the US simulation set-up.

Eight umbrella windows separated by 12 Å (position of ROS depicted by block crosses) were sampled in one simulation. In consecutive simulations, each species shifted by 0.12 Å. To sample the entire membrane system, 33 simulations were performed, yielding a total of 264 umbrella histograms from which a PMF was constructed.


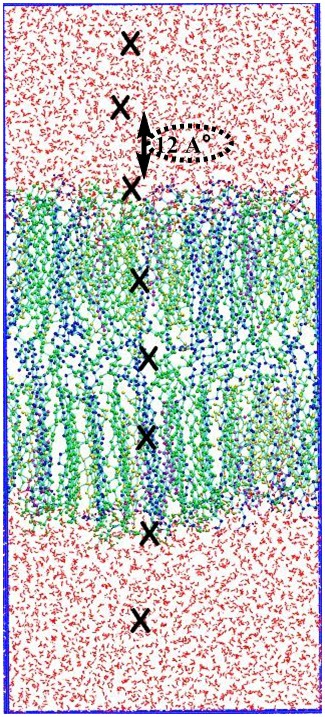

Supplement: Supplementary file 1 — Supplementary Dataset [file 41598_2019_40913_MOESM1_ESM.docx]
